# MD-Syn: synergistic drug combination prediction based on a multidimensional feature fusion method and attention mechanisms

**DOI:** 10.3389/fphar.2025.1564339

**Published:** 2025-07-14

**Authors:** XinXin Ge, Yi-Ting Lee, Shan-Ju Yeh

**Affiliations:** ^1^ School of Medicine, National Tsing Hua University, Hsinchu, Taiwan; ^2^ Institute of Bioinformatics and Structural Biology, National Tsing Hua University, Hsinchu, Taiwan; ^3^ Department of Life Science, National Tsing Hua University, Hsinchu, Taiwan

**Keywords:** drug combination, multidimensional feature fusion, graph neural network, attention mechanism, chemical language

## Abstract

Drug combination therapies have shown promising therapeutic efficacy in complex diseases and demonstrated the potential to reduce drug resistance. However, the vast number of possible drug combinations makes it difficult to screen them all in traditional experiments. Although computational models have been developed to address this challenge, existing methods often struggle to fully capture the complex biological interactions underlying drug synergy, limiting their predictive accuracy and generalization. In this study, we proposed MD-Syn, a computational framework based on a multidimensional feature fusion method and multi-head attention mechanisms. Given drug pair–cell line triplets, MD-Syn considers both one- and two-dimensional feature spaces simultaneously. It consists of a one-dimensional feature embedding module (1D-FEM), a two-dimensional feature embedding module (2D-FEM), and a deep neural network-based classifier for synergistic drug combination prediction. MD-Syn achieved an area under the receiver operating characteristic curve (AUROC) of 0.919 in five-fold cross-validation, outperforming the state-of-the-art methods. Furthermore, MD-Syn showed comparable results across four independent datasets. In addition, the multi-head attention mechanisms not only learn embeddings from different feature aspects but also focus on essential interactive feature elements, improving the interpretability of MD-Syn. In summary, MD-Syn is an interpretable framework to prioritize synergistic drug combination pairs using chemical and cancer cell line gene expression profiles. To facilitate broader community access to this model, we have developed a web portal (https://labyeh104-2.life.nthu.edu.tw/) that enables customized predictions of drug combination synergy effects based on user-specified compounds.

## Highlights

• We proposed MD-Syn, a novel computational framework for synergistic drug combination prediction based on multidimensional feature fusion methods and multi-head attention mechanisms, which achieved an area under the receiver operating characteristic curve (AUROC) of 0.919 in five-fold cross-validation.
• For the one-dimensional feature embedding module (1D-FEM) in MD-Syn, we applied a large-scale chemical language pre-trained model and a multi-layer perceptron classifier to obtain representations for small molecules and cancer cell lines, respectively.
• For the two-dimensional feature embedding module (2D-FEM) in MD-Syn, we used a graph convolutional network (GCN) to obtain the graph representation for each drug and leveraged the node2vec algorithm to learn the node embedding for each protein in the protein–protein interaction (PPI) network.
• We designed a trans-pooling block in the 2D-FEM with multi-head attention mechanisms that could not only capture representations from different feature aspects but also improve the interpretability of MD-Syn.
• By integrating two modalities of each input feature type, MD-Syn achieved a 2.6% improvement in AUROC for synergistic drug combination prediction, demonstrating the effectiveness of multidimensional feature integration.

## Introduction

Most human diseases are caused by complex biological processes that cannot be cured entirely by a single drug treatment strategy. Compared to single-agent therapies, drug combinations have the potential to improve efficacy, reduce host toxicity and side effects, and overcome drug resistance (Chou, 2006). The drug combination will present synergistic effects and may have antagonistic or additive effects (Foucquier and Guedj, 2015). In the clinical setting, synergistic effects may enable patients to be treated with a lower dose of each drug, resulting in fewer adverse side effects while still gaining the desired outcome, whereas antagonistic effects may cause patients to experience unexpected adverse side effects. Combination therapies have been explored to combat drug resistance, which cancer patients often encounter with single-agent treatments (Jin et al., 2023). Accurately predicting synergy and antagonism for drug–drug interactions (DDIs) is crucial for safer and improved patient prescriptions. However, the vast number of potential drug pairs makes it challenging to screen them all experimentally. In addition, the discovery of drug combination screening using traditional experimental methods would be very challenging in terms of time, cost, and efficiency. Therefore, developing computational methods to facilitate the discovery of synergistic drug combination therapies is needed.

Chemical fingerprints can describe specific properties of drugs, including substructure, related targets, and side effects, using a series of binary digits. Of note, natural language processing methods have been utilized on the Simplified Molecular Input Line Entry System (SMILES, chemical language), e.g., word2vec (Mikolov et al., 2013) and seq2seq (Xu et al., 2017). However, one-dimensional (1D) sequence data could not capture the spatial structure of molecules. In other words, the constructed models cannot learn structural information directly from the input data. To address the lack of spatial information, researchers have applied graph neural networks (GNNs) to obtain molecular graph representations with message passing (Gilmer et al., 2017). Our input graph-like data would consist of node (atom) features and an adjacency matrix. The idea is to update each node feature vector by aggregating the message vectors passed from its neighbor nodes along the edge of the graph. In this way, we can effectively obtain two-dimensional (2D) information of the drug.

The advent of high-throughput sequencing enables scientists to study cancer phenotypes from cancer omics, such as genomics or transcriptomics data. The omics data are also commonly used to construct cell line features for synergistic drug combination predictions. The cell line features play an indispensable role since a drug combination validated on one cell line may not be effective on another (Meng et al., 2010). Jeon et al. (2018) used mutations, copy number variations, and expression of genes in cancer-related pathways to depict cell line features, combined with pharmacological information to predict whether synergism or antagonism exists between two drugs. Celebi et al. (2019) leveraged multi-omics data and compound properties to build a machine learning model to predict anti-cancer drug combinations. Preuer et al. (2018) proposed a deep learning-based model, DeepSynergy, integrating chemical descriptors and cell line gene expression profiles to predict drug synergies. AuDNNsynergy applied three autoencoders to obtain gene expression, copy number variation, and mutation embeddings for individual cancer cell lines, combined with physicochemical features as input to a deep neural network that predicts the synergy score for pairwise drug combinations (Zhang T. et al., 2021). In addition, Yang et al. (2024) used drug characteristics such as 1D Morgan fingerprints and 3D atomic point cloud feature embeddings, along with cancer cell line attributes including gene expression and mutation data, to develop a multimodal deep learning model using bidirectional long short-term memory (Bi-LSTM) and gated multilayer perceptron (gMLP) networks for predicting synergistic anti-cancer drug combinations. These above-mentioned methods only consider extracting chemical property–cell line associations from one perspective but neglect a holistic view of interactions among features. Meanwhile, in the practical use of the model, it is sometimes difficult to obtain comprehensive information about the cell line, except for gene expression profiles. The protein–protein interaction (PPI) network is essential in physiological and pathological processes, including cell proliferation, differentiation, and apoptosis (Nero et al., 2014). The potential predictability of drug combinations by considering drug–drug and drug–disease relationships in the PPI has been demonstrated using the network-based approach (Cheng et al., 2019). Yang et al. (2021) proposed GraphSynergy, an adapted graph convolutional network (GCN) component, to encode the higher-order topological relationship in the PPI network of protein modules targeted by a pair of drugs and the protein modules associated with a specific cancer cell line (Yang et al., 2021). Based on GCN, PRODeepSyn integrates the PPI network with omics data to construct low-dimensional dense embedding for cell lines to predict anti-cancer synergistic drug combinations (Wang X. et al., 2022). However, there are still few studies considering the topological features of the drug and PPI network together in drug synergy prediction.

Attention mechanisms can improve prediction performance and enhance the interpretability of neural network structures (Wiegreffe and Pinter, 2019). For the graph-structured data, the graph attention network (GAT) introduces masked self-attention layers into the node feature propagation step and multi-head attention mechanisms to stabilize the learning process (Veličković et al., 2017). Moreover, the transformer, based solely on attention mechanisms, is beneficial to parallel computing, outperforming recurrent neural networks (RNNs) and convolutional neural networks (CNNs) (Vaswani et al., 2017). Instead of chemical information-based approaches, Liu and Xie (2021) used the target-based representation of drug molecules inferred from drug–target associations in the PPI network to implement TranSynergy based on attention mechanisms for synergistic prediction. Using the encoder of the transformer to learn drug features, DeepTraSynergy is a multitask prediction model that simultaneously considers synergy loss, toxic loss, and drug–target interaction loss during the training of a synergistic drug combination prediction model (Rafiei et al., 2023). Based on multi-head attention mechanisms, DTSyn, a dual-transformer encoder model, could capture different associations using a fine-granularity transformer encoder and a coarse-granularity transformer encoder for identifying novel drug combinations (Hu et al., 2022). It is noted that these proposed models have leveraged drug–target information as input, which may pose limitations when dealing with novel drugs whose molecular targets are unknown, thereby reducing their applicability in real-world experiments. Wang et al. (2023) implemented AttenSyn, which exploited the attention-based pooling module to learn interactive information between drug pairs to strengthen their representations in synergistic drug combination prediction without considering the biological network information. Assisted by multi-layer perceptron (MLP) and GAT, DeepDDS can capture gene expression patterns and chemical substructures for identifying synergistic drug combinations toward specific cancer cell lines (Wang J. et al., 2022). Instead of applying the transformer attention module, the attention mechanisms of GAT learn molecular graph representations, resulting in the loss of interactive information between drug pairs. AttentionDDI has been proposed to predict DDI based on a Siamese self-attention multi-model neural network that integrates multiple drug similarity measures (Schwarz et al., 2021). Combining drug feature representations into four different drug fusion networks, MDF-SA-DDI predicted DDI based on the auto-encoders with transformer self-attention mechanism (Lin et al., 2021). By fine-tuning a pre-trained language model, DFFNDDS applied a multi-head attention mechanism and a highway network to predict synergistic drug combinations (Xu et al., 2023). Monem et al. (2023) integrated multi-view graph data to build a multi-task learning model for simultaneously predicting a synergy score and a synergy class label. It should be noted that they applied multi-view learning to obtain the graph embedding, which involves taking the direct sum of the vector spaces corresponding to the views based on graph properties by considering different nodes and their possible paths. However, the computational time would increase with the complexity of graph data and also lead to the loss of edge features of molecular graphs. The above-mentioned studies have shown that attention mechanisms could improve not only the performance of models but also the interpretability ability in synergistic drug combination predictions. Meanwhile, those studies have demonstrated that using only 1D and 2D drug features leads to satisfactory prediction performance, but the integration of both dimension features together has been discussed less frequently.

**FIGURE 1:**
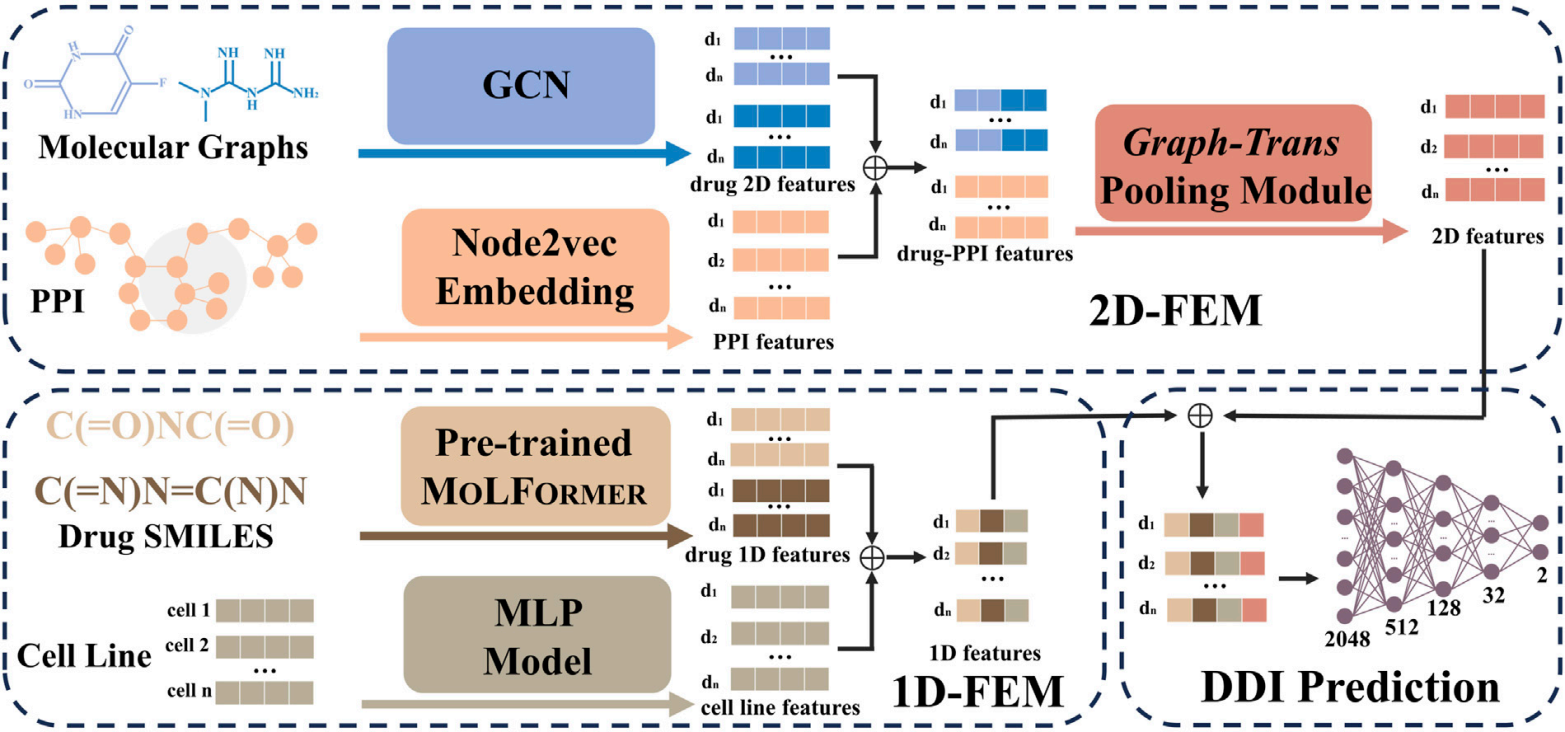
Architecture of MD-Syn. The computational framework of MD-Syn consists of a one-dimensional feature embedding module (1D-FEM), a two-dimensional feature embedding module (2D-FEM), and a fully connected neural network classifier for DDI prediction. For the 1D-FEM, molecular representations were obtained by fine-tuning MOLFORMER based on the chemical SMILES; the cell line representations were compressed using an MLP model. For the 2D-FEM, the molecular graph representations were learned using the GCN module, and the PPI representations were computed using the node2vec module. After combining both drug pair and PPI network 2D representations, they are further processed through a graph-trans pooling module with attention mechanisms to generate the final 2D features. Consequently, by concatenating the output from the 1D-FEM and 2D-FEM, we could utilize multidimensional features to train a neural network for synergistic drug combination prediction.

In this study, we propose MD-Syn, a novel framework that incorporates a multidimensional feature fusion method and multi-head attention mechanisms to predict synergistic drug combinations. As illustrated in Figure 1, MD-Syn merges the 1D and 2D representations of both drugs and proteins and feeds them into a fully connected neural network classifier to make a binary prediction (1: synergy; 0: antagonism). Comprehensive experiments have been designed and conducted on MD-Syn, showing its feasibility for synergy drug discovery and precision medicine practically.

## Materials and methods

### Dataset

The drug–drug interaction dataset was collected from the study by O’Neil et al. (2016). The dataset presented by O’Neil represents a comprehensive, unbiased, high-throughput screening of drug combinations, including 23,052 drug pairs, where each pair contains two chemicals and a cancer cell line. Among the dataset, there are 39 cancer cell lines across 7 different cancer types. The number of unique drugs was 38, which consists of 24 FDA-approved drugs and 14 experimental drugs (Preuer et al., 2018). The data preprocessing procedure followed the method described by Hu et al. (2022).The synergy score for each drug pair was calculated using the Combenefit tool (Di Veroli et al., 2016), which implements the well-established Loewe additivity model to assess whether a combination exhibits synergy or antagonism. The duplicated drug pairs were averaged as a unique drug pair. Considering class balancing, 10 is a threshold to classify drug pair–cell line triplets. Triplets with synergy scores higher than 10 were positive (synergistic) pairs, indicating stronger-than-additive effects, and those less than 0 were negative (antagonistic) pairs, representing less-than-additive combined effects. Hence, we obtained 13,243 unique triplets, including 6,188 positive pairs and 7,055 negative pairs that covered 38 unique drugs and 31 cancer cell lines. Moreover, the gene expression profiles of cancer cell lines are obtained from the Cancer Cell Line Encyclopedia (CCLE) (Ghandi et al., 2019). As our cancer cell line features, we took the landmark genes, which can cover 82% of the whole transcriptome information in the library of integrated network-based cellular signatures (LINCS) L1000 platform (Subramanian et al., 2017). Additionally, we considered four independent datasets—Oncology Screen (O’Neil et al., 2016), DrugCombDB (Liu et al., 2020), DrugComb (Zagidullin et al., 2019), and Merck (O’Neil et al., 2016)—to further validate the generalization ability of MD-Syn. All four independent datasets underwent the same data preprocessing workflow as the O’Neil dataset. A total of 1,919 drug synergy records were acquired for the Oncology Screen dataset, involving 21 unique drugs and 12 cell lines. In the DrugCombDB dataset, there are 36,626 drug combination records covering 358 drugs and 68 cell lines. The DrugComb dataset contains 74,924 drug combinations, including 10,643 synergistic and 64,281 antagonistic cases, spanning 1,221 unique experimental drugs and 53 cancer cell lines. The Merck dataset includes 12,411 drug combinations involving 36 unique drugs and 31 unique cell lines.

### Computational framework of MD-Syn

In this study, we take advantage of the multidimensional feature fusion method to build a synergistic drug combination prediction model, MD-Syn. The overall architecture of MD-Syn is shown in Figure 1. The network architecture mainly contains (1) a 1D-FEM, (2) a 2D-FEM, and (3) a fully connected neural network classifier for DDI prediction. We consider drug pairs and cell line features in two different dimensional views. After concatenating the representations generated by the 1D-FEM and 2D-FEM, we fed them into a fully connected neural network to predict synergistic drug combinations under a certain cell line. The details for each module are discussed in the following section.

### One-dimensional feature embedding module for drug pairs and cell lines

**FIGURE 2:**
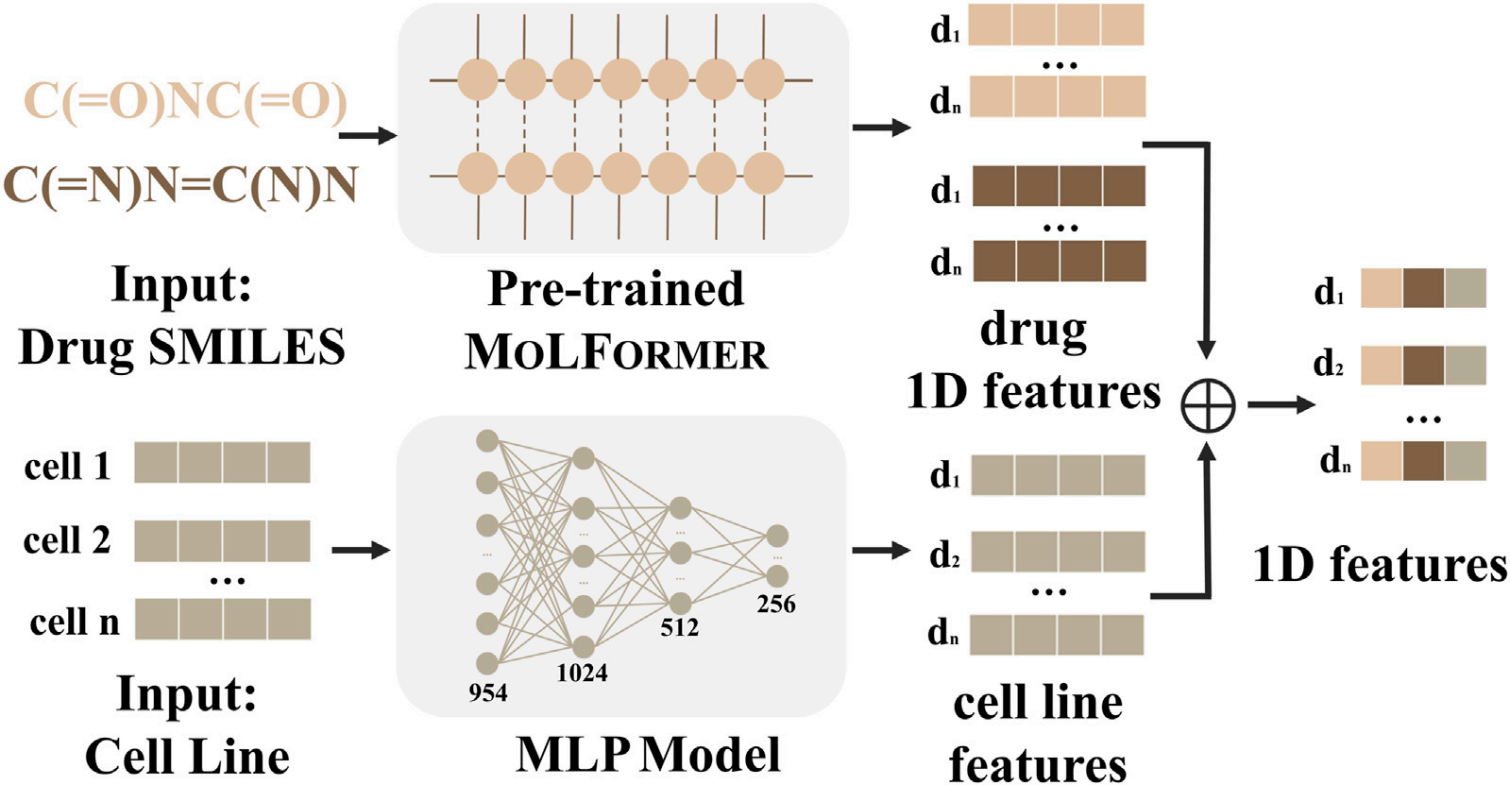
1D-FEM of MD-Syn. We leverage drug pair–cell line triplets to train MD-Syn. For one-dimensional drug features, we utilize a chemical language pre-trained model, MOLFORMER, to obtain each drug representation based on the SMILES strings. The cell line information is depicted using the 978 landmark genes in the LINCS L1000 platform. They are compressed using MLP. After concatenating both representations, we can obtain drug pair–cell line 1D features.

The 1D-FEM is presented in Figure 2. We first consider our input features, drug pair–cell line, in 1D view. Given the chemical SMILES for each drug, we leveraged MOLFORMER (Ross et al., 2022), which has been trained on over 1.1 billion molecules based on the transformer-based language model, to obtain chemical representations by fine-tuning the pre-trained model. The learned chemical representation from MOLFORMER would be a vector with 768 dimensions. For the cell line information in the drug pair–cell line triplet, based on the 978 landmark genes for each cancer cell line, we utilized three layers of MLP to gain the compressed embedding, resulting in 256 dimensions. After that, we merged the chemical representations of the drug pair, learned from MOLFORMER, with the compressed embedding of the corresponding cancer cell line as our 1D feature.

### Two-dimensional feature embedding module for drug pairs and cell lines

**FIGURE 3:**
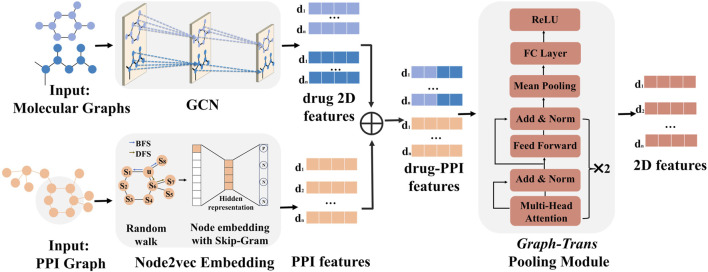
2D-FEM of MD-Syn. The training dataset of MD-Syn is based on drug pair–cell line triplets. We use a GCN to capture spatial information based on the molecular graph for each drug. Considering the PPI network, which comprises 978 landmark genes, we utilize the node2vec algorithm to obtain the embedding of each node. Given a drug pair–cell line triplet, we concatenate the 2D feature of drug pairs with PPI network embedding as the input of the graph-trans pooling module with a multi-head attention mechanism, which can generate more informative representations based on different feature aspects. We regard the output of the graph-trans pooling module as our used 2D features of the drug pair–cell line triplet.

To obtain the representation of the drug pair–cell line triplet in 2D view, we designed a 2D-FEM, including a GCN module, a node2vec module, and a graph-trans pooling module, as shown in Figure 3.

### Graph convolutional network in the 2D-FEM for molecular graph representation learning

Based on the RDkit (Landrum, 2013), we could convert the SMILE format of each drug to a molecular graph. A molecular graph could be represented as $G=\left(V,E\right)$ , where $V=\left\{v_{1},v_{2},\cdots ,v_{v}\right\}$ and E=V×V denote the node and edge sets, respectively. Let $X\in R^{V\times c}$ be the feature matrix of all nodes, where V is the number of nodes and c is the dimension of the node feature in a molecular graph, and $A\in R^{V\times V}$ be the adjacency matrix. We aim to learn the molecular graph representation. Given a molecular graph G , it consists of nodes (atoms) and bonds (edges). The inputs of the GCN include the node feature matrix $X\in R^{V\times c}$ and the adjacency matrix *A* $\in R^{V\times V}$ . The layer-wise propagation process is shown in Equation 1:

$$h^{\left(l+1\right)}=\sigma \left(D^{-\frac{1}{2}}AD^{-\frac{1}{2}}h^{\left(l\right)}W^{\left(l\right)}\right),$$ (1)

where A is the adjacency matrix of the molecular graph, ${\;D}_{ii}=\sum_{j}A_{ij}$ is a degree matrix, and $W^{l}$ is a layer-specific trainable weight matrix. $h^{l}\in R^{V\times d}$ is the matrix of activation, where d is the compressed dimension in the $l^{th}$ layer, and ${\;h}^{0}=X$ . $\sigma \left(\cdot \right)$ denotes the rectified linear unit (ReLU) activation function. In this study, we applied a two-layer GCN, followed by ReLU after each layer to capture the spatial information of the molecular graph in the GCN module.

### Node2vec in the 2D-FEM for protein–protein network representation learning

In this study, we constructed the PPI network (Oughtred et al., 2019), consisting of 978 landmark genes in LINCS L1000 (Subramanian et al., 2017). The nodes represent proteins, and the edges indicate biological associations between proteins. To obtain the representation of each node (protein), we applied the node2vec algorithmic framework (Grover and Leskovec, 2016), which could return feature representations that maximize the likelihood of preserving network neighborhoods of nodes in a low-dimensional feature space. Given our PPI network graph, the node2vec module in the 2D-FEM could assist us in obtaining a 128-dimensional feature representation for each node (protein).

### Graph-trans pooling with attention mechanisms in the 2D-FEM for drug pair–cell line representations

For the graph-trans pooling module in the 2D-FEM, we applied two transformer encoder layers with multi-head attention mechanisms, followed by a node mean pooling strategy and a fully connected layer to generate 2D representations, as shown in Figure 3. The input of the graph-trans pooling module is a matrix concatenated by molecular graph representation and PPI network embeddings. An attention function maps a query and a set of key–value pairs to an output, where the query, key, and value are all derived from the input matrix. In this study, instead of using a single attention function, we applied multi-head attention mechanisms, which project the queries, keys, and values h times using differently learned query, key, and value parameter matrices. For each of the projected processes, we project our input matrix X into a higher dimensional space to produce a query matrix, a key matrix, and a value matrix, as presented in Equations 2–4:

$$Q_{i}=XW_{i}^{Q},$$ (2)

$$K_{i}=XW_{i}^{K},$$ (3)

$$V_{i}=XW_{i}^{V},$$ (4)

where X denotes the input matrix. $W_{i}^{Q}$ , $W_{i}^{K}$ , and $W_{i}^{V}$ are the trainable parameter matrices of the i th head. $Q_{i}$ , $K_{i}$ , and $V_{i}$ stand for the query, key, and value matrices computed by the linear transformation of X for the i th head, respectively.

The output of the attention mechanisms for the *i*th head is described in Equation 5:

$$Attention\left(Q_{i},K_{i},V_{i}\right)=softmax\left(\frac{Q_{i}K_{i}^{T}}{\sqrt{d_{k}}}\right)V_{i},$$ (5)

where $d_{k}$ is the hidden dimensionality of the query and key. Meanwhile, the attention score matrix is the output of the softmax activation function. In this study, we used the multi-head attention function. There are four parallel attention layers (heads) to extract associations from different aspects. Therefore, we concatenate all heads and project them once again to obtain the final values, as shown in Equation 6:

$$Multihead\left(Q,K,V\right)=Concat\left({Head}_{1},{Head}_{2},{Head}_{3},{Head}_{4}\right)W^{O},$$ (6)

where ${Head}_{i}=Attention\left(Q_{i},K_{i},V_{i}\right)$ and $W^{O}$ is a trainable parameter matrix. It is noted that the output of the multi-head attention layer includes a residual connection, which may help mitigate the gradient vanishing problem, and is followed by layer normalization. In addition to attention sub-layers, each transformer encoder contains a two-layer feed-forward neural network. Hence, the transformer encoder output can be depicted as shown in Equation 7:

$${TransEnc}_{out}=\left(XW_{1}+b_{1}\right)W_{2}+b_{2},$$ (7)

where $W_{2}$ , $b_{1}$ , $W_{2}$ , and $b_{2}$ are trainable parameters. After going through two transformer encoder layers, we conducted node mean pooling, followed by a feed-forward neural network with the ReLU activation function to generate the 2D representation, which captures the spatial information of the molecular graph and the PPI network.

### Drug–drug interaction prediction for the synergistic effect of drug combination under specified cell lines

For predicting DDI with a synergistic effect, we built an MLP classifier, as shown in Figure 1. We regard the concatenation of the outputs from the 1D-FEM and 2D-FEM representations as $a^{0}$ , which serves as the input to the MLP classifier. The output of the last hidden layer of the MLP classifier is $y^{\prime }$ , which is shown in Equation 8:

$$y^{\prime }=W_{out}\cdot a^{l}+b_{out},$$ (8)

where $W_{out}$ and $b_{out}$ are the weight matrix and bias vector, respectively, whose values are updated using the backpropagation algorithm during the training process. $a^{l}$ is the output of the previous hidden layer, which is depicted in Equation 9:

$$a^{l}=\sigma \left(W^{l}a^{l-1}+b^{l}\right),$$ (9)

where $W^{l}$ and $b^{l}$ are the weight matrix and bias vector of the l th hidden layer, respectively. $\sigma \left(\cdot \right)$ is the ReLU activation function. Given the i th drug pair–cell line triplet, to compute the probabilities of the synergistic or antagonistic effect, there is a softmax function that follows the output of the last hidden layer, as shown in Equation 10:

$$y_{i}^{out}=\frac{e^{y_{i}^{\prime }}}{\sum_{j=1}^{K}e^{y_{j}^{\prime }}},$$ (10)

where K denotes the number of predicted classes (synergistic or antagonistic). Furthermore, for the training process of the MLP classifier, our goal is to minimize the cross-entropy loss. It is defined in Equation 11:

$$L=-\sum_{i=1}^{N}y_{i}\log\left(y_{i}^{out}\right),$$ (11)

where $y_{i}$ is the true label of the i th drug pair–cell line triplet and N is the total number of training samples.

## Result

### Experimental hyperparameter setup

MD-Syn is a computational framework that owns a significant amount of adjustable hyperparameters. It would be challenging to exhaustively explore all hyperparameter combinations. Thus, we decided to focus on several key hyperparameters and investigated their impacts on the MD-Syn performance of AUC in five-fold cross-validation. During the hyperparameter tuning process, we found that the learning rate had the largest impact on the MD-Syn performance (Supplementary Figure S1). It should be noted that all transformer encoder layers shared the same hyperparameters, including the number of attention heads and the feed-forward hidden layer size. The search space and the optimal hyperparameter settings are shown in Table 1.

**TABLE 1:** Hyperparameters of MD-Syn.

Hyperparameter	Value
GCN hidden unit	[78,128,128]; [78,256,128]; and **[78,512,128]**
Pooling method	Mean and max
Learning rate	1e-2; 1e-3; 1e-4; **5e-4**; 1e-5; and 1e-6
Dropout rate	No dropout; 0.1; 0.2; **0.3**; 0.4; 0.5
Number of attention heads	1; **2**; and 4
Type of activation function	ReLU and GELU
Number of transformer encoder layers	1; **2**; 3; and 4
Hidden size in the transformer encoder	32; **64;** and 128
Drug 1D feature embedding method	MOLFORMER and ChemBERTa

The bold values represent the optimal parameters.

### Performance comparisons between MD-Syn and baseline methods

To address the performance of MD-Syn, we compared MD-Syn with state-of-the-art methods and traditional machine learning models. Among the state-of-the-art methods, the deep learning-based models include DeepDDS (Wang J. et al., 2022) and DeepSynergy (Preuer et al., 2018), while the transformer-based model includes DTSyn (Hu et al., 2022). Moreover, traditional machine learning models include random forest (RF) (Breiman, 2001), XGBoost (Chen and Guestrin, 2016), and AdaBoost (Freund and Schapire, 1997). The experimental results of both state-of-the-art methods and traditional machine learning models were obtained using the same input datasets as MD-Syn. We followed the original parameter settings by referring to corresponding studies for state-of-the-art methods and used the default setting for the traditional machine learning models. Furthermore, for comparing the robustness of models, all the evaluation metrics for each method were computed based on five-fold cross-validation, as shown in Table 2. For MD-Syn, the average of the area under the receiver operating characteristic curve (AUROC), area under the precision–recall curve (AUPR), accuracy (ACC), true positive rate (TPR), and F1-score (F1) are 0.919, 0.910, 0.843, 0.840, and 0.833, respectively. The bar plot for performance comparisons between baseline methods and traditional machine learning-based models is shown in Supplementary Figure S2. MD-Syn outperformed other methods with the best evaluation metrics. Specifically, compared to the transformer-based model DTSyn, it achieves a 3.9% increase in AUROC, a 4.4% increase in AUPR, and a 4.9% increase in F1, which showed that MD-Syn had superior performance in predicting synergistic drug combinations.

**TABLE 2:** Performance comparisons of MD-Syn and baseline methods.

Method	AUROC	AUPR	ACC	BACC	PREC	TPR	KAPPA	F1
MD-Syn	**0.919 ± 0.005**	**0.910 ± 0.006**	**0.843 ± 0.007**	**0.843 ± 0.007**	**0.827 ± 0.009**	**0.840 ± 0.014**	**0.684 ± 0.014**	**0.833 ± 0.008**
DeepDDS	0.879 ± 0.021	0.86 ± 0.018	0.799 ± 0.023	0.797 ± 0.023	0.803 ± 0.019	0.757 ± 0.027	0.595 ± 0.046	0.779 ± 0.022
DTSyn	0.880 ± 0.004	0.866 ± 0.005	0.799 ± 0.008	0.798 ± 0.007	0.789 ± 0.011	0.779 ± 0.017	0.596 ± 0.015	0.784 ± 0.007
DeepSynergy	0.852 ± 0.007	0.841 ± 0.009	0.771 ± 0.008	0.771 ± 0.008	0.748 ± 0.014	0.779 ± 0.022	0.541 ± 0.016	0.763 ± 0.009
Random Forest	0.782 ± 0.006	0.822 ± 0.009	0.783 ± 0.006	0.782 ± 0.006	0.772 ± 0.010	0.760 ± 0.010	0.564 ± 0.013	0.766 ± 0.010
XGBoost	0.780 ± 0.008	0.822 ± 0.011	0.782 ± 0.007	0.780 ± 0.008	0.776 ± 0.022	0.752 ± 0.027	0.562 ± 0.015	0.763 ± 0.013
AdaBoost	0.761 ± 0.008	0.807 ± 0.007	0.764 ± 0.008	0.761 ± 0.008	0.763 ± 0.013	0.718 ± 0.021	0.524 ± 0.015	0.740 ± 0.009

The bold values represent the optimal parameters.

### Performance evaluation based on the leave-one-out cross-validation

In this study, we define “leave-one-out” as a group-level cross-validation strategy, in which an entire group of biologically relevant units (such as drug combinations, drugs, cell lines, or tissue types) is excluded one at a time from training and used for testing in a cross-validation setup. This differs from conventional leave-one-out cross-validation but allows us to systematically evaluate the generalization ability of MD-Syn across different biological inputs.

To assess the generalization ability of MD-Syn on unseen drug combinations, we performed a leave-drug-combination-out five-fold cross-validation. Specifically, we first counted the frequency of all unique drug combinations in our dataset and selected the top 15 most frequent combinations. These 15 drug combinations were grouped into five subsets, each containing three drug combinations. In each fold, one subset was left out for testing, while the other drug combinations were used for training. Moreover, other baseline methods were under the same data-splitting rule. As shown in Table 3, MD-Syn achieved the highest average AUROC, AUPR, and ACC scores of 0.865, 0.855, and 0.806, respectively, followed by DTSyn. Meanwhile, it can be observed that the deep learning-based and transformer-based models significantly outperformed traditional machine learning models.

**TABLE 3:** Performance evaluation based on leave-drug combination-out, leave-drug-out, leave-cell line-out, and leave-tissue-out experiments.

Method	Leave-drug combination-out			Leave-drug-out			Leave-cell line-out			Leave-tissue-out		
	AUROC	AUPR	ACC	AUROC	AUPR	ACC	AUROC	AUPR	ACC	AUROC	AUPR	ACC
MD-Syn	**0.865 ± 0.105**	**0.855 ± 0.098**	**0.806 ± 0.128**	**0.754 ± 0.068**	0.780 ± 0.269	0.666 ± 0.158	**0.804 ± 0.054**	**0.761 ± 0.056**	**0.737 ± 0.050**	**0.869 ± 0.016**	**0.852 ± 0.020**	**0.779 ± 0.018**
DeepDDS	0.801 ± 0.099	0.798 ± 0.188	0.592 ± 0.274	0.735 ± 0.058	0.767 ± 0.239	0.461 ± 0.174	0.802 ± 0.067	0.744 ± 0.074	0.724 ± 0.044	0.850 ± 0.013	0.830 ± 0.023	0.767 ± 0.014
DTSyn	0.809 ± 0.066	0.839 ± 0.173	0.727 ± 0.069	0.733 ± 0.055	0.775 ± 0.216	0.671 ± 0.117	0.792 ± 0.070	0.749 ± 0.062	0.704 ± 0.031	0.849 ± 0.014	0.832 ± 0.019	0.750 ± 0.028
DeepSynergy	0.748 ± 0.099	0.792 ± 0.231	0.708 ± 0.095	0.662 ± 0.045	0.732 ± 0.056	0.462 ± 0.028	0.774 ± 0.075	0.733 ± 0.072	0.712 ± 0.043	0.842 ± 0.009	0.826 ± 0.017	0.758 ± 0.011
Random Forest	0.596 ± 0.108	0.835 ± 0.101	0.738 ± 0.06	0.644 ± 0.052	**0.786 ± 0.199**	0.621 ± 0.149	0.714 ± 0.056	0.742 ± 0.052	0.720 ± 0.054	0.780 ± 0.015	0.822 ± 0.022	0.780 ± 0.015
XGBoost	0.627 ± 0.136	0.827 ± 0.137	0.771 ± 0.048	0.654 ± 0.058	**0.790 ± 0.191**	0.644 ± 0.122	0.711 ± 0.054	0.739 ± 0.049	0.717 ± 0.053	0.768 ± 0.014	0.810 ± 0.020	0.767 ± 0.014
AdaBoost	0.612 ± 0.120	0.788 ± 0.195	0.740 ± 0.059	0.630 ± 0.045	0.775 ± 0.204	**0.685 ± 0.110**	0.672 ± 0.041	0.706 ± 0.043	0.677 ± 0.041	0.708 ± 0.014	0.762 ± 0.024	0.708 ± 0.015

The bold values represent the optimal parameters.

In addition, the exclusion of drug combinations during training does ensure that MD-Syn has not encountered particular pharmaceuticals. Therefore, we conducted a leave-drug-out five-fold cross-validation experiment to evaluate the model’s predictive ability on unseen drugs based on the multidimensional feature representations learned from previously seen drugs. Here, we selected the top five most frequently appearing drugs, namely, BEZ-235, dasatinib, MK-8669, bortezomib, and erlotinib, in our dataset. For each fold of the training process, we only removed one out of the top five drugs. Drug pair–cell line triplets containing the specified drug would be put into the testing set, and the remaining drug pair–cell line triplets would be in the training set. In Table 3, for MD-Syn, the average values of AUROC, AUPR, and ACC are 0.754, 0.780, and 0.666, respectively. Meanwhile, we find that RF, XGBoost, and AdaBoost perform slightly better on average in terms of AUPR and ACC in the leave-drug-out experiment. However, MD-Syn still shows acceptable prediction performance. Specifically, considering the top five most frequent drugs in our dataset, MD-Syn achieved the best predictive performance on erlotinib with an AUROC value of 0.813 (Supplementary Table S2), indicating the model’s ability to maintain strong performance on specific drugs when sufficient data are available.

To access the generalization capability on unseen cell lines, we also performed leave-cell-line-out five-fold cross-validation experiments. Similarly, based on the cell line counts in our dataset, we selected the top five cell lines, namely, CAOV-3, LNCaP, MSTO, T47D, and XR751, which appeared most frequently to form five separate testing sets. In each training fold, we selected the drug pair–cell line triplets that do not belong to the designated cell line as the training set, while the remaining triplets are used as the testing set. The average values of AUROC, AUPR, and ACC are 0.804, 0.761, and 0.737, respectively (Table 3). These values indicate that MD-Syn exhibits a generalization capability to previously unseen cell lines. In particular, analyzing the five most prevalent cell lines in our dataset, MD-Syn demonstrated superior predictive performance on CAOV-3 with an AUROC value of 0.878 (Supplementary Table S3), implying the model’s capacity to sustain robust performance on specific cell lines when adequate data are present.

Furthermore, we evaluated MD-Syn under more rigorous scenarios by performing leave-tissue-out five-fold cross-validation experiments. In particular, we selected the top five tissue types that appeared most frequently and sequentially eliminated all the cell lines associated with one of them. The top five tissue types are the lung, skin, intestine, ovary, and breast. During the training process, we sequentially consider each of the top five tissues as the testing set. Compared to baseline methods, MD-Syn has the highest average AUROC and AUPR scores of 0.869 and 0.852, respectively (Table 3). Moreover, MD-Syn and other baseline methods hold a better prediction performance in intestine-correlated drug combinations within these five tissue types (Supplementary Figure S3).

### Performance evaluation on independent datasets

To further evaluate the generalization ability, we used the dataset proposed by O’Neil et al. (2016) to train MD-Syn and baseline methods and then leveraged the Oncology Screen (O’Neil et al., 2016), DrugCombDB (Liu et al., 2020), DrugComb (Zagidullin et al., 2019), and Merck (O’Neil et al., 2016) as the independent datasets for model validation. In Table 4, MD-Syn has the highest AUROC scores of 0.967 and 0.625 for the Oncology Screen and DrugCombDB datasets, respectively. Compared to other deep learning-based and transformer-based methods, MD-Syn demonstrated its better generalization abilities on external datasets.

**TABLE 4:** Performance evaluation metrics for independent datasets.

Method	Oncology screen			DrugCombDB			Merck			DrugComb		
	AUROC	AUPR	ACC	AUROC	AUPR	ACC	AUROC	AUPR	ACC	AUROC	AUPR	ACC
MD-Syn	**0.967**	**0.968**	**0.891**	**0.625**	**0.638**	**0.582**	0.604	0.6	0.56	0.586	0.146	0.776
DeepDDS	0.900	0.907	0.804	0.578	0.582	0.541	**0.996**	**0.994**	**0.985**	**0.702**	0.238	0.434
DTSyn	0.921	0.92	0.841	0.609	0.635	0.575	0.5	0.746	0.507	0.5	**0.545**	**0.91**

The bold values represent the optimal parameters.

We also conducted an overlap analysis between the training dataset (O’Neil) and the four independent datasets. The Oncology Screen dataset shares 71.3% of its entries with the training set. DrugCombDB has an overlap of approximately 0.3%, while DrugComb and Merck datasets show a 0% overlap. These findings help explain the relatively higher performance observed on the Oncology Screen and underscore the importance of evaluating non-overlapping datasets to assess true generalization. Although DeepDDS performed better in the Merck and DrugComb datasets, this may be attributed to a higher similarity between its training data and these two datasets, potentially leading to an advantage during evaluation.

### Model ablation study

MD-Syn takes multidimensional feature representations into account. The architecture of MD-Syn comprises the 1D-FEM and 2D-FEM. The 2D-FEM contains a graph-trans pooling module that introduces transformer encoder layers with multi-head self-attention mechanisms. To comprehensively investigate the contribution of each module, we conducted the ablation study for the following different combinations of modules: (1) MD-Syn with the 1D-FEM only, (2) MD-Syn with the 2D-FEM only, and (3) MD-Syn with the 2D-FEM without the graph-trans pooling module. The corresponding results of evaluation metrics for synergistic drug combination prediction are shown in Table 5.

**TABLE 5:** Results of evaluation metrics in the ablation study.

Method	AUROC	AUPR	ACC	BACC	PREC	TPR	KAPPA	F1
MD-Syn	**0.919 ± 0.005**	**0.910 ± 0.006**	**0.843 ± 0.007**	**0.843 ± 0.007**	**0.827 ± 0.009**	**0.840 ± 0.014**	**0.684 ± 0.014**	**0.833 ± 0.008**
MD-Syn-1D-FEM	0.893 ± 0.005	0.878 ± 0.006	0.809 ± 0.006	0.810 ± 0.005	0.783 ± 0.015	0.819 ± 0.021	0.671 ± 0.010	0.800 ± 0.007
MD-Syn-2D-FEM	0.846 ± 0.010	0.827 ± 0.017	0.764 ± 0.015	0.765 ± 0.021	0.734 ± 0.019	0.776 ± 0.073	0.528 ± 0.038	0.754 ± 0.042
MD-Syn-2D-FEM-without g*raph-trans*	0.824 ± 0.005	0.808 ± 0.014	0.748 ± 0.010	0.743 ± 0.010	0.779 ± 0.037	0.649 ± 0.054	0.489 ± 0.020	0.706 ± 0.020

The bold values represent the optimal parameters.

In MD-Syn with the 1D-FEM only (MD-Syn-1D-FEM), we utilized the large-scale chimerical pre-trained model MOLFORMER (Ross et al., 2022) to obtain 1D representation for each drug based on its SMILES. The cell line information was depicted through 978 landmark genes, compressed using an MLP. After concatenating these 1D representations, the later MLP classifier module would predict synergistic drug combinations. The overall evaluation metrics are shown in Table 5. The average AUROC, AUPR, ACC, and F1 values are 0.893, 0.878, 0.809, and 0.800, respectively. MD-Syn with the 2D-FEM only (MD-Syn-2D-FEM) focused on using 2D representations generated using the GCN and node2vec algorithms based on molecular graphs and the PPI network comprising 978 landmark genes, respectively. MD-Syn-2D-FEM achieved average AUROC, AUPR, ACC, and F1 scores of 0.846, 0.827, 0.764, and 0.754, respectively. Furthermore, the last variant combination is the 2D-FEM without the graph-trans pooling module (2D-FEM without graph-trans). The performance of the 2D-FEM without graph-trans is inferior to the MD-Syn-2D-FEM, indicating that multi-head self-attention mechanisms facilitate MD-Syn to capture different feature aspects, resulting in better model performance. Moreover, by incorporating 2D information from the input feature type, the performance of MD-Syn improves by 2.6% in AUROC. The multidimensional feature representation consideration leads us to achieve higher evaluation metrics in synergistic drug combination prediction. In summary, the ablation study of these three variants in five-fold cross-validation demonstrates the importance of each component in MD-Syn and the effectiveness of graph-trans pooling in the 2D-FEM.

### Interpretation of MD-Syn based on hidden embeddings and attention scores

**FIGURE 4:**
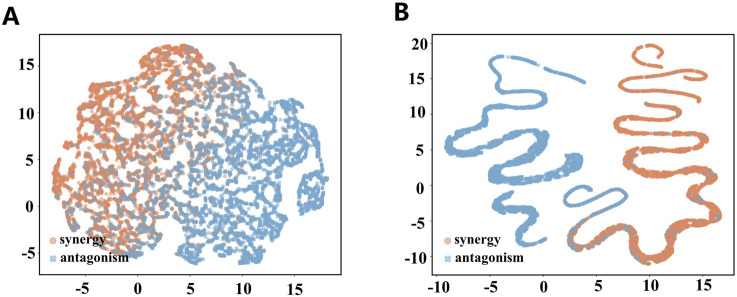
Visualization of hidden embeddings using UMAP. **(A)** 512-dimensional hidden layer embeddings visualized using UMAP for synergistic and antagonistic drug pair–cell line triplets. **(B)** 32-dimensional hidden layer embeddings visualized using UMAP for synergistic and antagonistic drug pair–cell line triplets.

The DDI prediction module in MD-Syn is a fully connected neural network responsible for performing binary classification of the drug pair–cell line triplets as either synergistic or antagonistic. To understand whether our DDI prediction module truly learns some patterns based on the integrated multidimensional feature representations, we extracted outputs generated from 512 and 32 hidden layers. After that, we utilized a dimension reduction algorithm, UMAP (McInnes et al., 2018), to project our embedding into 2D space for each drug pair–cell line triplet (Figure 4). After the training process, we found that synergistic and antagonistic drug pairs were distinctly separated into two clusters. In other words, MD-Syn could identify the differences between synergistic and antagonistic effects of drug pair–cell line triplets. This phenomenon offers evidence showing that MD-Syn is capable of making synergistic drug combination predictions.

The 2D-FEM contains a graph-trans pooling module built by two transformer encoder layers using multi-head self-attention mechanisms. We analyzed the attention scores for each atom in the molecular graph and each gene in the PPI network. In this study, we used a synergy drug combination of MK-2206 with MK-8669 in the cell line MDA-MB-436 and an antagonism drug combination of 5-fluorouracil (5-FU) and sorafenib in the cell line HT29 as examples. MK-2206 is a potent allosteric inhibitor of AKT, a serine/threonine kinase that plays a central role in the PI3K/AKT/mTOR pathway. Aberrant activation of AKT signaling is frequently observed in various human cancers, including breast, lung, and prostate cancers, contributing to enhanced tumor growth, survival, and therapeutic resistance. MK-2206 inhibits the phosphorylation of AKT isoforms (AKT1/2/3), effectively shutting down pro-survival signals (Xu et al., 2013). MK-8669 is a selective inhibitor of mTOR, which is a critical downstream effector of PI3K/AKT signaling. mTOR drives protein synthesis, metabolic adaptation, and angiogenesis, processes that are often hyperactivated in cancers. MK-8669 directly inhibits mTORC1, suppressing the phosphorylation of S6 and 4EBP1, thereby reducing cellular proliferation and tumor growth (Xu et al., 2013). The combination of MK-2206 and MK-8669 addresses the limitations of monotherapy by providing a dual blockade of the PI3K/AKT/mTOR pathway. MK-2206 prevents the feedback reactivation of AKT induced by MK-8669, leading to a more comprehensive suppression of tumor-promoting pathways. In WU-BC4 and WU-BC5 xenograft models, this combination significantly reduced tumor proliferation and angiogenesis, with a pronounced effect observed in PTEN-deficient tumors, which are particularly reliant on PI3K/AKT signaling (Xu et al., 2013). Furthermore, 5-FU is a cornerstone chemotherapeutic agent used for decades in the treatment of various solid tumors, including colorectal, gastric, breast, and head and neck cancers. It primarily acts as an antimetabolite by inhibiting thymidylate synthase (TS), thereby blocking DNA synthesis and leading to tumor cell death. 5-FU also incorporates into RNA and DNA, further impairing cellular functions (Longley et al., 2003). Sorafenib is a multi-kinase inhibitor approved for the treatment of advanced renal cell carcinoma, hepatocellular carcinoma, and differentiated thyroid cancer. It targets multiple tyrosine kinases involved in tumor angiogenesis (VEGFR and PDGFR) and cell proliferation (RAF kinases). In Caco-2 cell line *in vitro* experiment, 5-FU and sorafenib showed antagonism, which exhibited pathway divergence and potential physical interference (Wehler et al., 2013).

**FIGURE 5:**
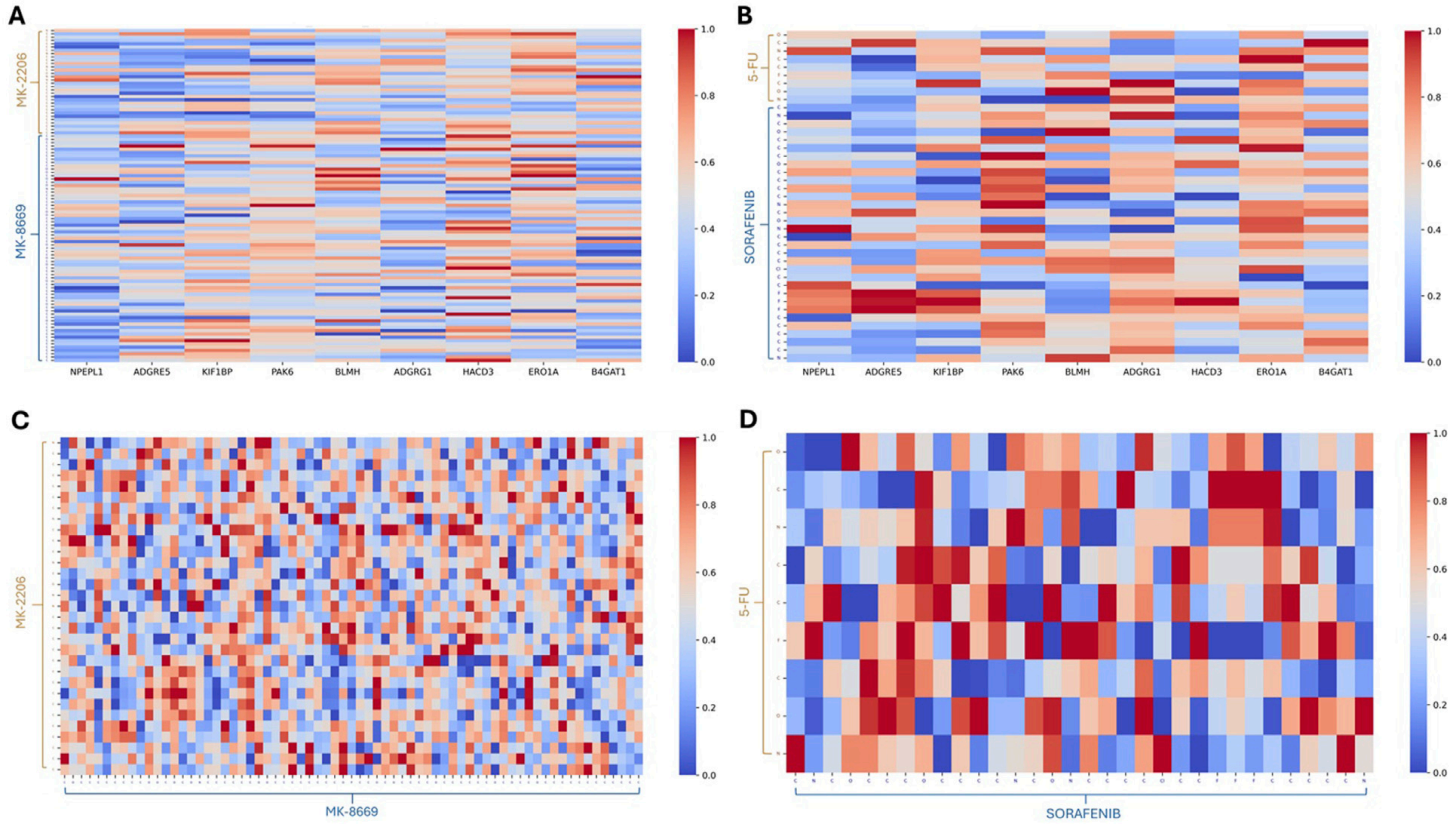
Heatmaps based on the attention scores. **(A)** Heatmap for the drug combination of MK-2206 and MK-8669 versus essential genes based on the attention scores. **(B)** Heatmap for the drug combination of 5-FU and sorafenib versus essential genes based on the attention scores. **(C)** Heatmap for MK-2206 versus MK-8669 within the drug combination pair based on the attention scores. **(D)** Heatmap for 5-FU versus sorafenib within the drug combination pair based on the attention scores.

After obtaining the attention score matrix based on multi-head self-attention mechanisms, we performed min–max normalization along columns. According to the learned attention score, we further aim to investigate how the essential genes in the PPI network affect synergistic and antagonistic drug combinations. For the synergistic combination of MK-2206 and MK-8669, MK-2206 exhibited higher attention weight toward PAK6, ERO1A, and HACD3, while MK-8669 exhibited high attention on BLMH, PAK6, and HACD3. Furthermore, PAK6, ERO1A, HACD3, and BLMH are functionally associated with protein PI3K/AKT signaling (Huang et al., 2022; Yang et al., 2020), ER stress (Liu et al., 2023), lipid metabolism (Wang et al., 2024), and protein degradation (Okamura et al., 2011), respectively. Notably, PAK6 received intense attention from both MK-2206 and MK-8669, suggesting that it may act as a convergent effector downstream of the PI3K/AKT/mTOR signaling pathway (Figure 5A). In contrast, the antagonistic combination of 5-FU and sorafenib demonstrated distinct attention patterns. 5-FU showed strong attention to ERO1A and BLMH, implicating its involvement in oxidative stress and protein degradation pathways. These may reflect ER stress and proteostasis disruption triggered by nucleoside analog toxicity. Meanwhile, sorafenib highlighted genes such as *PAK6*, *ADGRG1*, and *ERO1A*. PAK6, a serine/threonine kinase involved in cytoskeletal regulation and MAPK signaling, showed sharply elevated attention from specific sorafenib atoms, suggesting direct pathway convergence. ADGRG1, a G-protein-coupled receptor associated with cell migration (Zhang S. et al., 2021), also received considerable localized attention. As ERO1A is involved in ER oxidative folding and redox homeostasis, its relevance may reflect a stress-related mechanism; therefore, the overlap in attention on ERO1A may not reflect complementarity but rather independent activation of ER stress under distinct regulatory contexts. Specifically, 5-FU-induced activation of ER stress is primarily driven by nucleoside misincorporation into RNA and DNA, leading to proteostasis disruption and unfolded protein response (UPR). In contrast, sorafenib likely induces ER stress indirectly through the inhibition of kinase pathways and modulation of cell survival signals. Such conflicting downstream signaling, particularly across MAPK, ER stress, and GPCR-mediated migration axes, may underlie the observed antagonism between these two agents (Figure 5B).

Functional group interactions have been recognized as influential factors in drug synergy and antagonism (Yin et al., 2014; Lee et al., 2013). To interpret how molecule structure influences predicted synergism or antagonism, we traced back atom-level attention weights to the corresponding chemical substructures. We specifically focused on regions of high attention intensity in the atom–atom interaction heatmaps, identifying key functional groups likely responsible for pharmacodynamic interactions. Molecular structures with atom index annotation are included in Supplementary Figure S4. In the synergy pair MK-2206 and MK-8669, a highly focused region was identified between atoms C:20–30 of MK-2206 and C:10–20 of MK-8669 (Figure 5C). The corresponding atoms on MK-2206 form a substituted phenyl ring adjacent to the triazolopyrimidine core, while those on MK-8669 largely constitute an ester moiety. The elevated attention in this region suggests a non-covalent interaction hot spot. In contrast, this ester region is not associated with a specific high-affinity target; its chemical flexibility and polarity may allow MK-8669 to engage MK-2206’s aromatic system adaptively, enhancing the binding orientation. In contrast, for the antagonistic pair 5-FU and sorafenib, intense cross-attention was detected between 5-FU’s C:2–N:3 segment and sorafenib’s trifluoromethyl group (C:22–F:23, F:24, and F:25) (Figure 5D). These regions correspond to the electron-rich pyrimidinone nitrogen in 5-FU and the strongly electron-withdrawing CF_3_ moiety in sorafenib. Both structures possess high polarity and electronic activity, which may lead to electrostatic repulsion or steric hindrance, potentially contributing to the observed antagonistic effect between 5-FU and sorafenib. This atomic-level attention mapping chemically interprets drug synergy and antagonism, linking structural motifs to functional outcomes. Together, these multiscale analyses demonstrate how MD-Syn’s attention mechanisms can reveal mechanistically relevant patterns in synergistic and antagonistic interactions, offering insights beyond performance metrics.

### MD-Syn: a web portal to predict the synergistic effect of drug combinations based on chemical structures and cancer cell line gene expression profiles

To make it available to the public, we have developed a web portal based on Shiny for Python, enabling users to predict the synergistic effects of drug combinations through a web interface. The platform processes the input drugs against the model’s training data using MD-Syn, generating results for 1,178 drug synergy combinations: 38 (the number of O'Neil dataset unique drugs) × 31 (the number of cancer cell lines) = 1,178. Upon completion of MD-Syn computation, the web interface returns combination results specific to the user-selected cell lines, providing predictions of synergistic or antagonistic effects for each drug combination. To utilize the platform, users must first obtain SMILES notation for their selected drugs from chemical databases such as PubChem or ChEMBL. Users then input this information and other required data (drug name and job title) into the web portal’s information fields and select their target cell line from among 31 cancer cell lines for prediction using the MD-Syn model. The prediction results are displayed in a comprehensive table showing drug combinations, cell lines, and whether their interactions are synergistic or antagonistic. For more detailed analysis, users can examine the drug synergy probability distribution interactive plot, which offers features such as adjustable displays of drug combinations through a slider bar and visualization download options. Detailed instructions for using the interactive plot can be found in the Interactive Plot Features section of the website. Users can receive comprehensive results containing all 1,178 predictions through email if a valid email address is provided. For those requiring assistance with the procedure, detailed step-by-step instructions for using MD-Syn are available on the website. We believe that this user-friendly MD-Syn web portal will effectively accelerate wet laboratory drug screening processes and significantly contribute to advances in drug discovery.

## Discussion

Drug combinations offer a more efficient therapeutic strategy. Dealing with numerous drug combinations, computational methods would be a faster and cheaper alternative in assisting the development of combination therapies. Although numerous computational methods have been developed to predict synergistic drug combinations, there are still limitations in fully capturing the complex biological interactions underlying drug synergy. For example, DeepDDS utilizes graph structures and gene expression profiles to predict drug–drug interactions through concatenated embeddings and fully connected networks. However, it does not explicitly capture fine-grained biological interactions between molecular substructures, genes, and cell lines. DTSyn improves upon this problem by introducing dual-transformer encoders to model both fine-granularity features (substructure-gene) and coarse-granularity features (drug–cell line). However, it focuses solely on coarse- or fine-level representations and may not comprehensively integrate multiple biological modalities. To address these limitations, we introduce a novel computational framework, MD-Syn, which leverages multidimensional feature representations for drug combination prediction. MD-Syn incorporates a 1D-FEM and a 2D-FEM to integrate learned drug and cell line features across different modalities. Using a large-scale pre-trained model, the 1D-FEM learned the drug representations from their chemical language and obtained the embeddings for cancer cell lines from CCLE genomic profiling using an MLP. Moreover, in the 2D-FEM, we learned graph representation for each drug using a GCN and obtained node embeddings from the PPI network using the node2vec algorithm. Our findings from the ablation study (Table 5) showed that integrating two modalities—sequence and structural data—improved overall performance in drug combination prediction compared to using a single feature modality alone. Meanwhile, multi-head self-attention mechanisms in the graph-trans pooling module facilitate the interpretability ability of MD-Syn. By the multi-head self-attention mechanisms, MD-Syn could capture representations from different aspects of relationships among drug pair–cell line triplets.

In addition to the primary evaluations, we conduct further experiments to strengthen the analysis of the MD-Syn design. First, we investigated whether graph isomorphism networks (GINs) could provide a better representation of the PPI network compared to the random walk-based embeddings originally used in MD-Syn. Despite GIN’s expressive capacity, the experimental results revealed that our original random walk-based approach consistently outperformed the GIN-based method across all evaluation metrics (Supplementary Table S1). It demonstrated that random walk-based embeddings can better capture the relevant biological signals within the PPI network under the MD-Syn framework. Furthermore, we explored the impact of expanding the training data scale using a larger DrugComb dataset. After retraining MD-Syn on the DrugComb data, the framework achieved an average AUROC value of 0.845, an accuracy of 0.768, and an F1 score of 0.523 (Supplementary Figure S5). The substantial class imbalance inherent in the DrugComb dataset may have limited improvement in AUC, resulting in slightly lower predictive performance than models trained on the O'Neil dataset. Additionally, we compared different sources of cell line gene expression features by substituting CCLE gene expression profiles with gene perturbation profiles from the LINCS L1000 dataset. When using LINCS-derived profiles, MD-Syn achieved an average AUROC value of 0.906, an accuracy of 0.837, and an F1 score of 0.847 under five-fold cross-validation (Supplementary Figure S6). However, our observations showed that models trained on CCLE-derived expression profiles consistently achieved slightly better predictive performance than those trained on LINCS perturbation profiles. These results suggest that using CCLE gene expression profiles provides a biologically robust and reliable foundation for accurate synergy prediction within the MD-Syn framework.

Although MD-Syn has demonstrated outstanding performance compared to state-of-the-art methods, our proposed model has some limitations. First, the training dataset that MD-Syn learned was trained on is based on the Loewe score. However, there are several distinguished methods to compute expected drug combination effects from experimental data, such as the combination index (CI)–isobologram equation (Huang et al., 2019), Bliss (Demidenko and Miller, 2019), ZIP score (Yadav et al., 2015), and Loewe score (Lederer et al., 2018). The calculated drug synergy scores would not be the same or consistent based on different quantification methods. To further improve the data quality, it is necessary to develop a new data correction method to incorporate different datasets. Second, the attention-based method surely provides us with a way to interpret MD-Syn. However, the atom-level coding method toward small molecules may limit the chemical interpretation. To address this limitation, merging the function-level coding method for compounds may enhance our understanding of the underlying factors that influence synergistic or antagonistic effects. Furthermore, we found that incorporating multidimensional or multi-modal input feature types leads to improved performance in synergistic drug combination prediction. Hence, integrating 3D conformation information of compounds and proteins into the graph-based model will be part of our future work.

## Conclusion

In summary, MD-Syn is an innovative framework for synergistic drug combination prediction that integrates multidimensional feature representation through the 1D-FEM and 2D-FEM. It is noted that MD-Syn demonstrated significant improvements in model performance compared to state-of-the-art methods, achieving an AUROC value of 0.919 in five-fold cross-validation experiments. Additionally, the framework offers model interpretability via multi-head attention mechanisms, which identify key molecular and cellular factors contributing to synergy prediction. MD-Syn not only advances our current understanding of drug synergy prediction but also lays a solid foundation for future developments in computational drug combination discovery and precision medicine. Beyond cancer treatment, MD-Syn’s architecture exhibits potential adaptability to other complex diseases, such as neurodegenerative disorders, where drug synergy plays an essential role in therapeutic advancement. Furthermore, the model’s flexible design shows promise for broader applications, including drug–target binding affinity prediction, hence extending its impact on drug discovery.

## Data Availability

All the data used in this study were from the online database. Drug combination data were from O'Neil dataset (O’Neil et al., 2016). Cancer cell line genomic data were downloaded from the DepMap portal (https://depmap.org/portal). The PPI network information was from Biogrid (Oughtred et al., 2019). The source code of MD-Syn and processed data used in this research are applicable upon request.
