# Supplementary material for: MD-Syn: synergistic drug combination prediction based on a multidimensional feature fusion method and attention mechanisms
Source: Front Pharmacol. 2025 Jul 14;16:1564339. doi: 10.3389/fphar.2025.1564339 (PMC12301336; doi:10.3389/fphar.2025.1564339)
Supplement: Supplementary file 1 [file DataSheet1.docx]

**Supplementary Materials**

**PPI network embedding methods validation**

We conducted an additional experiment using Graph Isomorphism Network (GIN)[1], a powerful variant of graph neural networks (GNNs) that has been shown to be theoretically as expressive as the Weisfeiler-Lehman graph isomorphism test. GIN uses sum aggregation and a learnable MLP to distinguish graph structures, making it particularly well-suited for tasks where subtle topological differences matter, such as capturing differential gene interactions across cancer cell lines. To model the cellular context of drug combinations, we constructed protein-protein interaction (PPI) networks using interaction data from the STRING database [2], which integrates evidence from multiple sources, including experiments and computational predictions. For each cancer cell line, we constructed a graph where nodes represent 978 landmark genes (from CCLE expression profiles) and edges represent STRING-derived interactions among these genes. Each cell line was represented as a distinct graph, with node features corresponding to the normalized expression values of that specific cell line. The graph was then processed using GIN, which performs sum-based message passing followed by an MLP, allowing the model to capture intricate topological patterns specific to each cell line.

Despite GIN’s expressive capacity, the experimental results in the Supplementary Information (SI) revealed that our original method, using node2vec-based embeddings for the PPI network, consistently outperformed the GIN-based representations across all evaluation metrics (Table S1). This suggests that the node2vec-based approach better captures the relevant biological signals in our current modeling setup, possibly due to its ability to preserve global graph topology with greater efficiency and its compatibility with our downstream architecture. These results validate our current choice of PPI network embedding and indicate that while GNN-based modeling is a promising direction, the random walk-based approach remains more suitable for our task under the present experimental setting.

**MD-Syn model evaluation based on DrugComb dataset**

We retrained our proposed model, MD-Syn, on DrugComb [3], a dataset that is larger than the primary dataset used in our study. In DrugComb, the results of drug combination screening studies are accumulated and standardized. In data preprocessing for DrugComb, we converted each drug’s chemical structure, denoted as a simplified molecular input line entry system (SMILES) string, into a molecular graph using RDKit [4], in which atoms are regarded as nodes and bonds as edges for graph representation learning in MD-Syn architecture. To generate binary classification labels for drug combinations, we adopted the Loewe synergy score: combinations with scores greater than 10 were labeled as synergistic (label 1), while those with scores less than 0 were labeled as antagonistic (label 0). After that, we could obtain 118367 drug combinations across 288 cancer cell lines, including 10643 synergistic pairs and 107724 antagonistic pairs. Noted that DrugComb dataset exists class imbalanced problem, with a roughly 10:1 ratio of antagonistic to synergistic drug combinations. This class imbalanced problem would severely hinder the learning performance of standard classification models, often biasing predictions toward the majority class. To address this problem, we retained all 10643 synergistic pairs and randomly sampled 50% of the antagonistic pairs, resulting in a more balanced dataset of 74924 drug combinations. Among these, 10643 were labeled as synergistic and 64281 as antagonistic, including 1221 unique experimental drugs and 53 cancer cell lines. By doing so, the down sampling step could help us to generate a more balanced dataset that can better support the learning of both synergistic and antagonistic patterns. To further mitigate the effects of class imbalance and enhance the model’s sensitivity to minority class (synergy) samples, we employed the Focal Loss [5] during training. Focal loss is specifically designed to focus learning on hard-to-classify examples by dynamically scaling the cross-entropy loss. It down-weights easy (majority class) examples and places more emphasis on difficult (minority class) cases, thus enabling the model to better distinguish synergistic drug combinations. This strategy has proven effective in domains with extreme label imbalance, such as object detection. After training based on the processed DrugComb dataset, the MD-Syn framework achieved the average of AUROC, accuracy, and F1 being 0.845, 0.768, and 0.523, respectively, as shown in Figure S5. The limited improvement in AUC may be attributed to the substantial class imbalance that remains in the DrugComb dataset.

**MD-Syn model evaluation based on gene perturbation profiles from the LINCS L1000 dataset**

We conducted an additional analysis to validate the biological interpretability of our model outputs by incorporating experimentally derived gene perturbation profiles. Specifically, we initially curated 13,243 drug combination pairs from the O'Neil drug combination database. To integrate transcriptional perturbation information, we matched compound and cell line pairs to available gene expression profiles from the LINCS L1000 dataset. After matching, a total of 2,933 drug combination samples spanning 38 unique drugs and 7 cell lines were retained for model development.

The LINCS L1000 profiles capture transcriptional changes induced by compound treatments and thus serve as a proxy for drug-induced gene perturbations within our model framework. While we have not yet explicitly incorporated external perturbation datasets such as CRISPR or RNAi screens, the inclusion of LINCS-derived gene expression signatures represents an initial step towards integrating experimental perturbation evidence.

Our model demonstrated strong predictive performance when utilizing gene perturbation profiles from the LINCS L1000 dataset, achieving an average AUROC of 0.906, ACC of 0.837, and F1-score of 0.847 under 5-fold cross-validation (Figure S6). Nevertheless, we observed that using CCLE-derived baseline cell line expression profiles yielded even slightly better performance compared to LINCS-derived perturbation profiles. These findings suggest that our current modeling framework, based on CCLE expression features, possesses strong predictive power and biological relevance, providing a reliable foundation for accurate synergy prediction.

**Reference**

1. Xu, K., et al., *How powerful are graph neural networks?* arXiv preprint arXiv:1810.00826, 2018.

2. Szklarczyk, D., et al., *STRING v11: protein–protein association networks with increased coverage, supporting functional discovery in genome-wide experimental datasets.* Nucleic acids research, 2019. **47**(D1): p. D607-D613.

3. Zheng, S., et al., *DrugComb update: a more comprehensive drug sensitivity data repository and analysis portal.* Nucleic Acids Res, 2021. **49**(W1): p. W174-W184.

4. Bento, A.P., et al., *An open source chemical structure curation pipeline using RDKit.* J Cheminform, 2020. **12**(1): p. 51.

5. Lin, T.-Y., et al. *Focal loss for dense object detection*. in *Proceedings of the IEEE international conference on computer vision*. 2017.


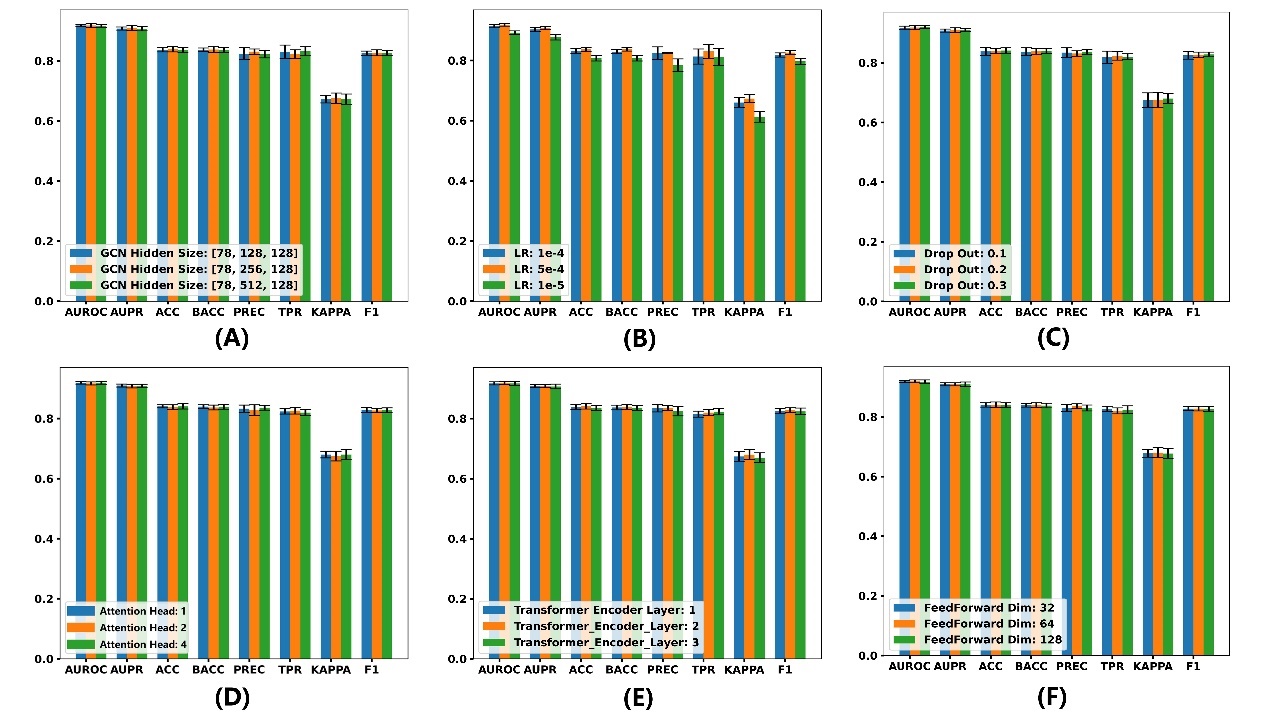


**Figure S1.** Hyperparameter tuning results in five-fold cross-validation. (A) The hidden layer size tuning of graph-convolutional network for MD-Syn. (B) The learning rate tuning results for MD-Syn. (C) The dropout rate tuning results for MD-Syn. (D) The attention head tuning results for MD-Syn. (E) The transformer encoder layer tunning results for MD-Syn. (F) The feedforward classifier layer tuning results for MD-Syn.


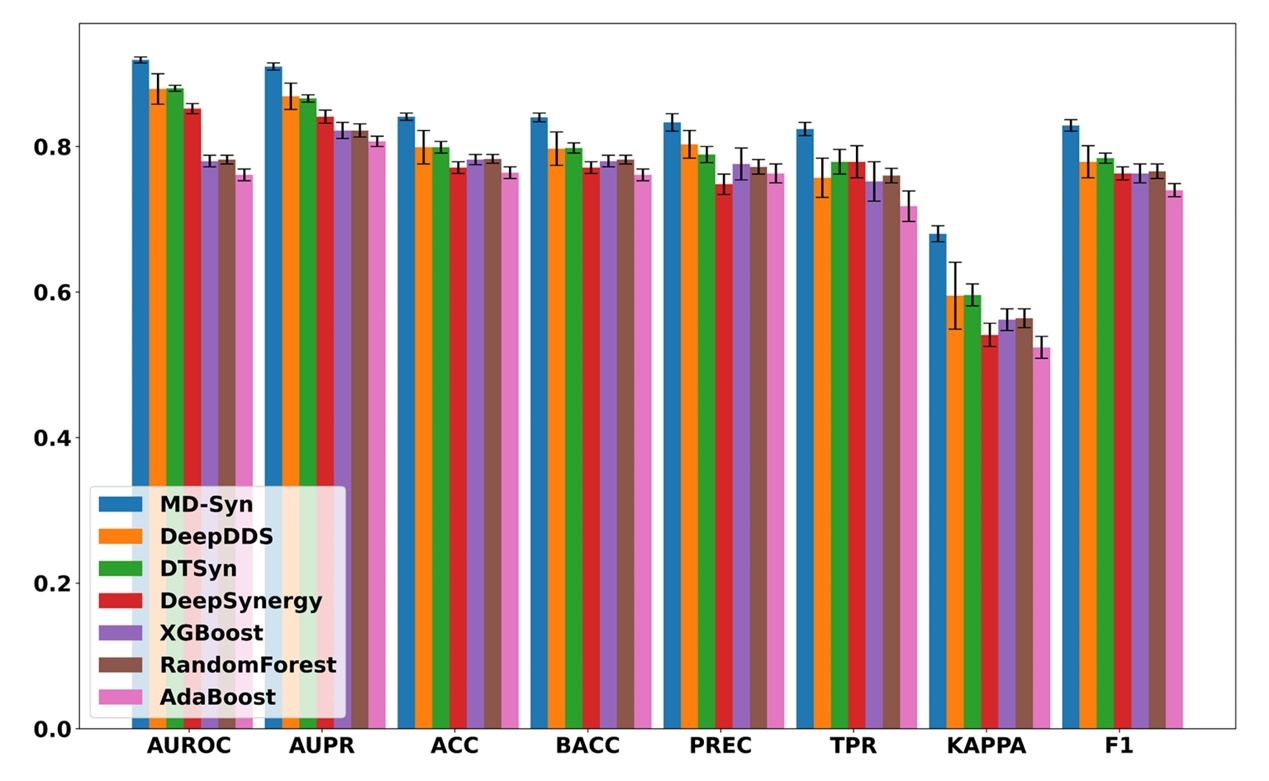


**Figure S2.** The bar plot for performance comparisons between baseline methods and traditional machine learning-based models.


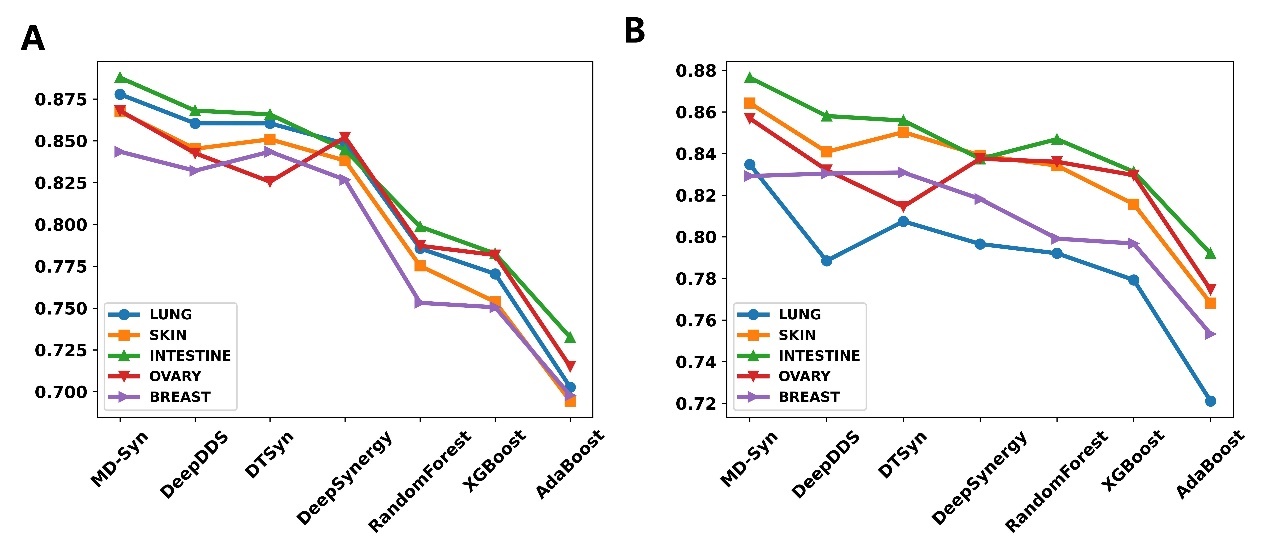


**Figure S3.** Performance results of leave-tissue-out. (A) Performance results of the average under the receiver operating characteristic curve (AUROC) for MD-Syn and baseline methods based on the top five tissue types including lung, skin, intestine, ovary, and breast. (B) Performance results of the average area under the precision-recall curve (AUPR) for MD-Syn and baseline methods based on the top five tissue types including lung, skin, intestine, ovary, and breast.


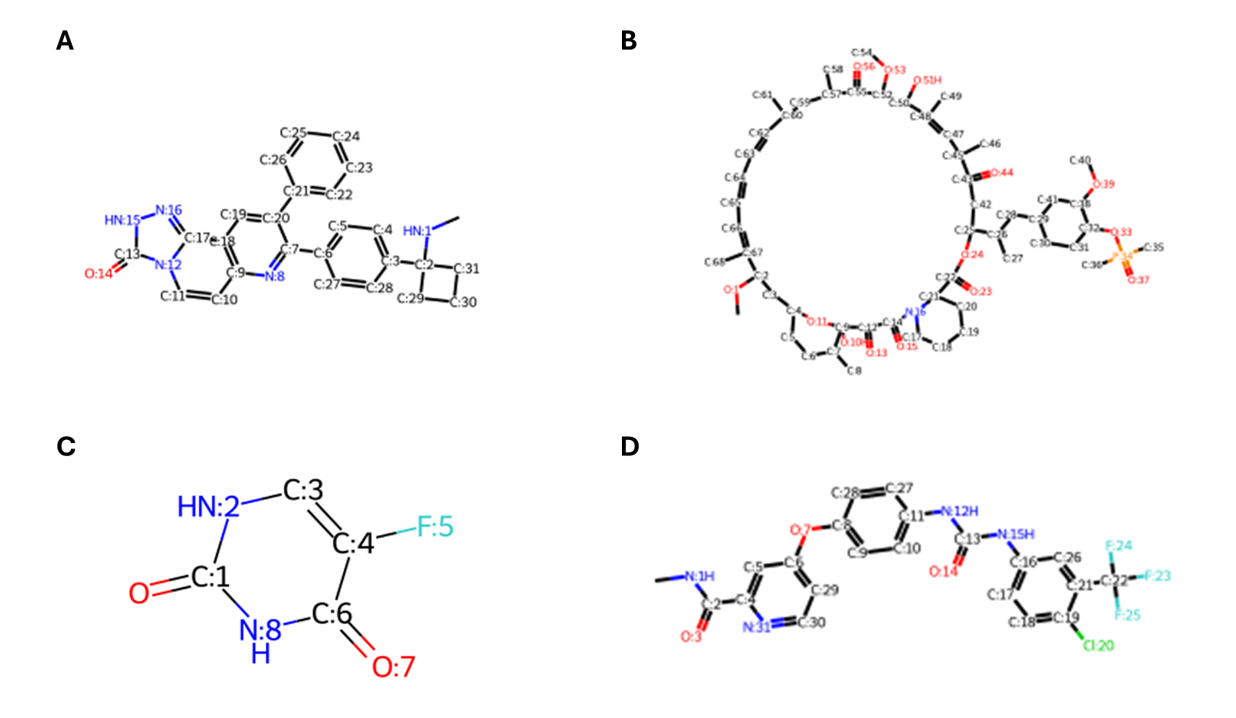


**Figure S4.** Molecular structures with atom index annotation of the drug compounds.

(A) MK-2206, an allosteric AKT inhibitor, featuring a triazolopyrimidine core with aromatic substituents. (B) MK-8669, a macrolide-derived mTOR inhibitor, is characterized by a large lactone ring and multiple ester/phosphoester groups. (C) 5-Fluorouracil (5-FU), a fluorinated pyrimidine analog with a fluorine-substituted uracil ring. (D) Sorafenib, a multi-kinase inhibitor bearing a trifluoromethyl-substituted aromatic moiety and a urea linker. Atom index were used to map attention scores back to specific atomic regions, enabling the identification of functional-group-level interaction hotspots in structural interpretability analysis.


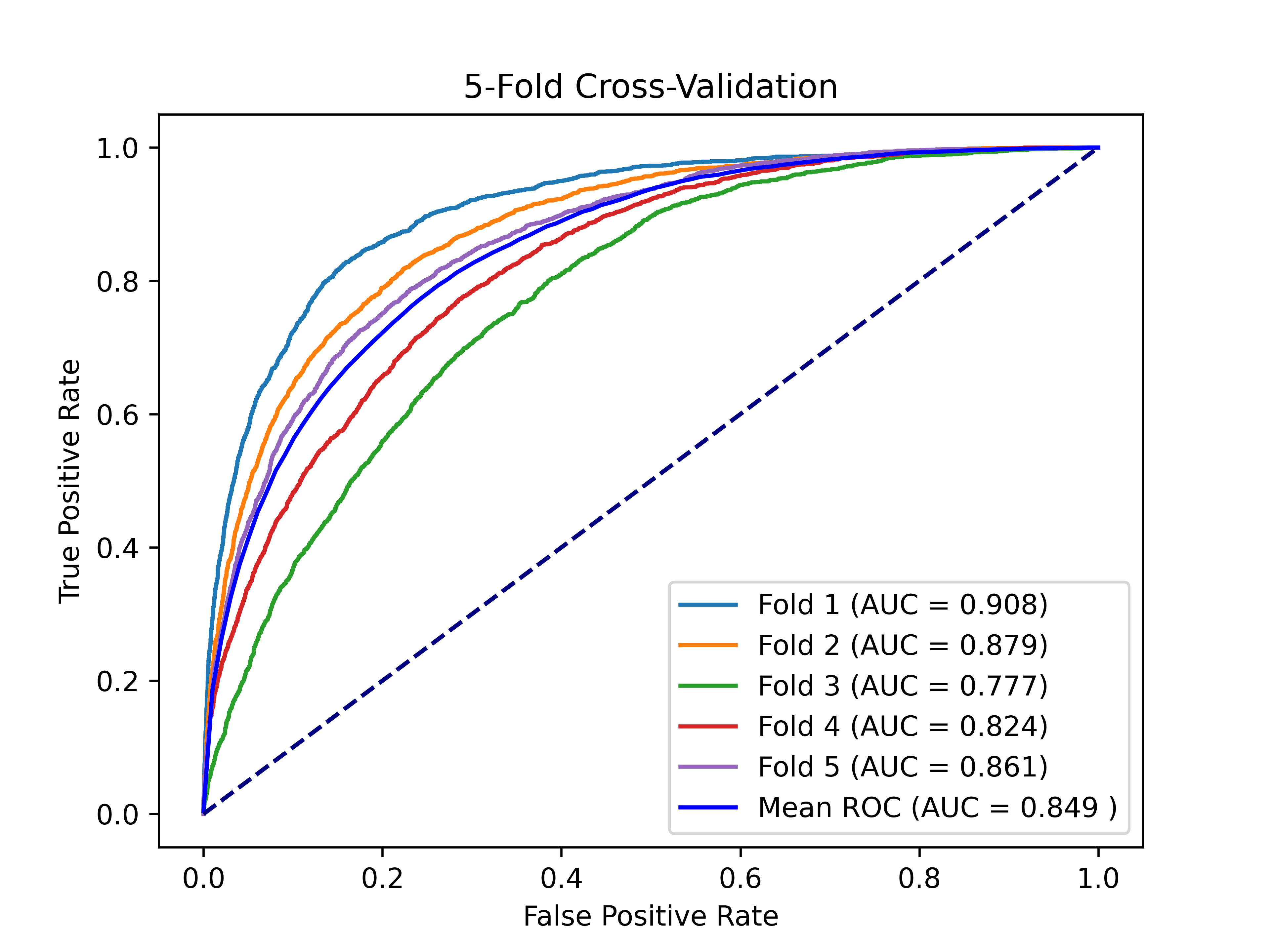


**Figure S5.** 5-fold cross-validation on the DrugComb dataset with focal loss. The dataset is partitioned into five stratified folds, maintaining the original class distribution (synergy vs. antagonism) in each fold. In every iteration, four folds are used for training and one for validation. The model is trained using the focal loss function to address class imbalance.


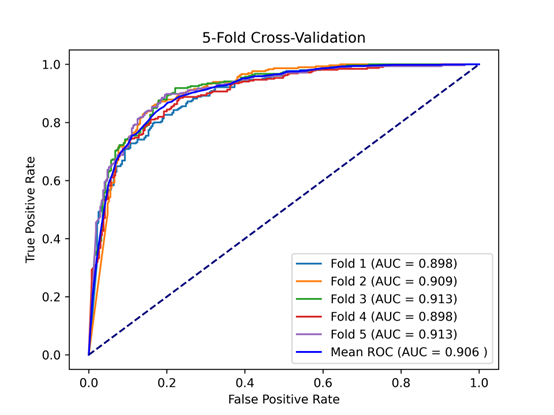


**Figure S6.** Model performance on the O'Neil drug combination dataset using 5-fold cross-validation. Gene expression profiles of cell lines were concatenated from the LINCS database.

**Table S1.** Comparison of PPI Network Embedding Methods: Node2vec vs. GIN

| **Methods** | **AUROC** | **AUPR** | **ACC** | **F1** |
| --- | --- | --- | --- | --- |
| **GIN** | 0.73158 | 0.67562 | 0.67424 | 0.66026 |
| **Node2vec** | **0.919** | **0.91** | **0.843** | **0.833** |

This table presents the performance comparison between two different PPI network embedding strategies as two-dimensional features of cell lines: (1) random walk-based node2vec embeddings and (2) graph neural network-based Graph Isomorphism Network (GIN) embeddings.

**Table S2.** Performance metrics from leave-drug-out cross-validation on the top five most frequent drugs.

| **Leave-drug-out** | **AUROC** | **AUPR** | **ACC** | **F1** |
| --- | --- | --- | --- | --- |
| **leave-BEZ235-out** | 0.802 | 0.966 | 0.764 | 0.853 |
| **leave-DASATINIB-out** | 0.660 | 0.814 | 0.428 | 0.357 |
| **leave-MK-8669-out** | 0.792 | 0.946 | 0.597 | 0.692 |
| **leave-BORTEZOMB-out** | 0.703 | 0.311 | 0.830 | 0.271 |
| **leave-ERLOTINIB-out** | 0.813 | 0.861 | 0.710 | 0.723 |

This table shows the performance metrics (AUROC, AUPR, ACC, F1-score) for MD-Syn in leave-drug-out cross-validation on the top five most frequent drugs. MD-Syn achieved the best performance on Erlotinib (AUROC = 0.813, AUPR = 0.861).

**Table S3.** Performance metrics from leave-cell-line-out cross-validation on the top five most frequent cell lines.

| **Leave-cell-out** | **AUROC** | **AUPR** | **ACC** | **F1** |
| --- | --- | --- | --- | --- |
| **leave-CAOV3-out** | 0.878 | 0.848 | 0.782 | 0.788 |
| **leave-LNCAP-out** | 0.746 | 0.691 | 0.695 | 0.645 |
| **leave-MSTO-out** | 0.740 | 0.707 | 0.673 | 0.664 |
| **leave-T47D-out** | 0.810 | 0.772 | 0.753 | 0.694 |
| **leave-ZR751-out** | 0.847 | 0.786 | 0.782 | 0.667 |

This table shows the performance metrics (AUROC, AUPR, ACC, F1-score) for MD-Syn in leave-cell-line-out cross-validation on the top five most frequent cell lines. MD-Syn achieved the best performance on CAOV3 (AUROC = 0.878, AUPR = 0.848).
